# A machine learning-based treatment prediction model using whole genome variants of hepatitis C virus

**DOI:** 10.1371/journal.pone.0242028

**Published:** 2020-11-05

**Authors:** Hiroaki Haga, Hidenori Sato, Ayumi Koseki, Takafumi Saito, Kazuo Okumoto, Kyoko Hoshikawa, Tomohiro Katsumi, Kei Mizuno, Taketo Nishina, Yoshiyuki Ueno

**Affiliations:** 1 Department of Gastroenterology, Yamagata University Faculty of Medicine, Yamagata, Japan; 2 Genome Informatics Unit, Institute for Promotion of Medical Science Research, Yamagata University, Yamagata, Japan; 3 School of Nursing, Yamagata University Faculty of Medicine, Yamagata, Japan; Nihon University School of Medicine, JAPAN

## Abstract

In recent years, the development of diagnostics using artificial intelligence (AI) has been remarkable. AI algorithms can go beyond human reasoning and build diagnostic models from a number of complex combinations. Using next-generation sequencing technology, we identified hepatitis C virus (HCV) variants resistant to directing-acting antivirals (DAA) by whole genome sequencing of full-length HCV genomes, and applied these variants to various machine-learning algorithms to evaluate a preliminary predictive model. HCV genomic RNA was extracted from serum from 173 patients (109 with subsequent sustained virological response [SVR] and 64 without) before DAA treatment. HCV genomes from the 109 SVR and 64 non-SVR patients were randomly divided into a training data set (57 SVR and 29 non-SVR) and a validation-data set (52 SVR and 35 non-SVR). The training data set was subject to nine machine-learning algorithms selected to identify the optimized combination of functional variants in relation to SVR status following DAA therapy. Subsequently, the prediction model was tested by the validation-data set. The most accurate learning method was the support vector machine (SVM) algorithm (validation accuracy, 0.95; kappa statistic, 0.90; F-value, 0.94). The second-most accurate learning algorithm was Multi-layer perceptron. Unfortunately, Decision Tree, and Naive Bayes algorithms could not be fitted with our data set due to low accuracy (< 0.8). Conclusively, with an accuracy rate of 95.4% in the generalization performance evaluation, SVM was identified as the best algorithm. Analytical methods based on genomic analysis and the construction of a predictive model by machine-learning may be applicable to the selection of the optimal treatment for other viral infections and cancer.

## Introduction

In recent years, machine-learning has expanded to include about 200 learning algorithms developed to enable relevant responses to the “big data” of the rapidly evolving scientific community of today [1]. In general, machine-learning is a method for deriving the optimal combination in a mathematical model from a vast number of explanatory variables (feature quantity) based on information on outcomes such as treatment results. While machine-learning is difficult to grasp compared with conventional statistical methods, it is very useful for analyzing big data because the combination of optimal explanatory variables is automatically obtained [2]. In addition, deep learning [3], which is known as the next generation of machine-learning, has been developed, and a learning method that identifies new outcomes using learning models has been developed, which is expected to be used for machine-learning-based medical treatment.

In recent years, the development and implementation of next-generation sequencing (NGS) technology has made genomic sequencing cost-effective. For instance, we can now easily sequence and analyze the RNA sequence of the entire genome of hepatitis C virus (HCV). With regard to HCV treatment, the advent of direct-acting antiviral drugs (DAA) has led to dramatically improved treatment outcomes, and more patients can now obtain a sustained virological response (SVR) [4]. However, as treatment-resistant variants emerge, some cases remain in which the virus is not eliminated. Although DAA treatment has a high therapeutic effect because of its direct action on the HCV, the likelihood of obtaining SVR depends on the location of the variation in the HCV genome [5]. It is well documented that a variety of viral quasispecies are found in patients with chronic HCV infection [6], but there are also non-SVR cases that cannot be explained only by the known resistance mutations.

In this study, we analyzed full-length HCV genomes obtained using NGS technology, developed a preliminary prediction system using several machine-learning models based on variants of the HCV genome, and evaluated the generalization performance.

## Genomics materials and methods

### Sampling and method overview

**Fig 1:**
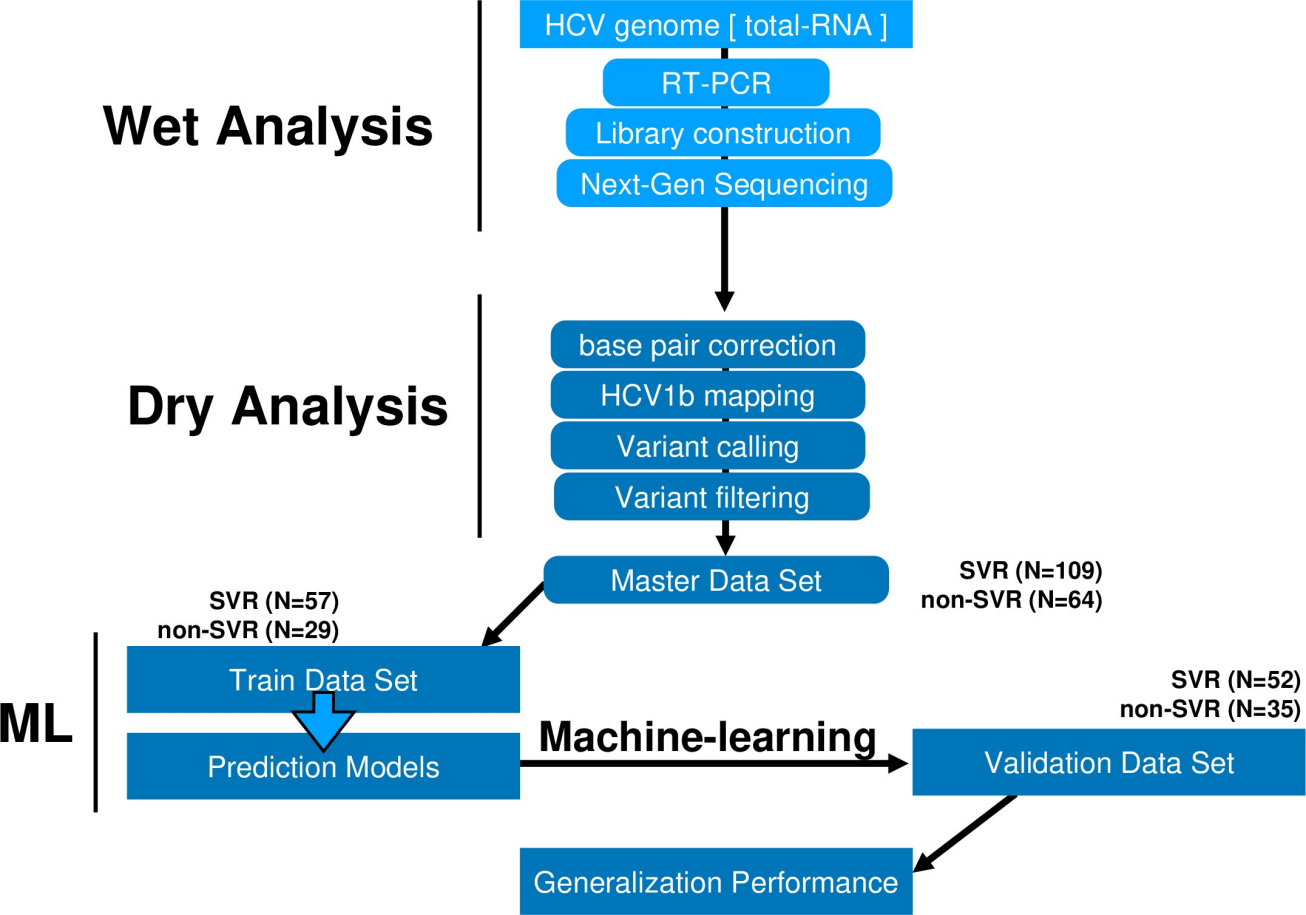
Study workflow.

Between October 2014 and September 2018, informed consent was obtained in writing from all patients with hepatitis C, prior to enrollment in this study. Blood samples were collected from each patient who had given consent to enable the extraction of HCV RNA. The workflow of the study is displayed in Fig 1. Blood samples were collected at Yamagata University Hospital and clinics located in Yamagata Prefecture in North-West Japan from HCV patients before DAA treatment. The study was approved by the Ethical Review Committee of Yamagata University Faculty of Medicine (IRB 2019–254) and was conducted according to the principles of the Declaration of Helsinki.

The study group comprised 109 patients successfully obtaining SVR at 24 weeks (SVR) and 64 with SVR24 failure (non-SVR) (Table 1). HCV genomic RNA was extracted from the 173 patient sera before DAA treatment, full-length HCV genome sequences were obtained using NGS technology, and sequence data were mapped to the HCV 1b reference sequence (NCBI accession ID AJ238799, D90208, M58335). Next, we performed variant calling and filtering and developed a Variant Call Format. The variants were digitized and used as the master data set for down-stream analyses. The 109 SVR HCV genomes and 64 non-SVR genomes were randomly divided into a train-data set and a validation-data set. A total of 57 SVR and 29 non-SVR genomes (training data set) were subject to nine machine-learning algorithms and used to develop the prediction model. Using the prediction model, the accuracy of identification was evaluated using 52 SVR and 35 non-SVR genomes (evaluation data set) (Fig 1 and Table 1).

**Table 1:** Patient characteristics.

	Training-Data Set			Validation-Data Set		
	SVR Mean (SD) n = 57	non-SVR Mean (SD) n = 29	*p value*	SVR Mean (SD) n = 52	non-SVR Mean (SD) n = 35	*p value*
**Age (years)**	**65.75 (11.09)**	**67.58 (7.88)**	0.78*	**69.04 7.93)**	**67.46 (10.05)**	0.55*
**Sex (male/female)**	**9/20**	**25/32**	0.25**	**12/23**	**20/32**	0.69**
**Platelet count (×10**^**4**^**/μL)**	**14.15 (7.38)**	**30.74 (54.13)**	0.38*	**16.44 (6.73)**	**27.7 (40.82)**	0.45*
**Aspartate aminotransferase (U/L)**	**60.09 (30.99)**	**56.42 (30.53)**	0.44*	**57.65 (36.89)**	**59.7 (35.25)**	0.83*
**Alanine aminotransferase (U/L)**	**55.93 (42.56)**	**50.46 (57.35)**	0.27*	**56.37 (40.79)**	**62.17 (53.41)**	0.88*
**Albumin (g/dL)**	**3.98 (0.44)**	**4.08 (0.28)**	0.53*	**4.06 (0.40)**	**4.04 (0.34)**	0.87*
**Total bilirubin (mg/dL)**	**0.83 (0.28)**	**0.77 (0.23)**	0.42*	**0.82 (0.33)**	**1.03 (0.82)**	0.42*
**HCV RNA (log IU/mL)**	**6.23 (0.49)**	**6.45 (0.62)**	0.09***	**6.41 (0.52)**	**6.56 (0.46)**	0.17***
**DCV+ASV/SOF+LDV/SOF+LDV+RBV**	**29/28/0**	**22/5/2**		**30/22/0**	**28/6/1**	**0.95**

DCV: Daclatasvir, ASV: Asunaprevir, SOF: Sofosbuvir, LDV: Ledipasvir, RBV: Ribavirin,

* Wilcoxon test (Mann–Whitney U-test),

** Chi-square test,

*** One-way analysis of variance (ANOVA).

#### Extraction of HCV rRNA and cDNA synthesis

For the extraction of RNA from the HCV viral genome, 500 μL of the serum supernatant including HCV viral particles was concentrated in a collection tube by centrifugation at 120,000 rpm at 4°C for 1.5 h. HCV viral genomic RNA was extracted using the QIAmp Viral RNA kit (Qiagen) following the recommendations of the manufacturer [7]. After extraction, the viral RNA was stored at –80°C until use.

Full-length HCV genome cDNA was synthesized by Reverse-Transcription PCR with polyA primers and Superscript™ III reverse transcriptase (Thermo Fisher Scientific), and then subsequently amplified by multiplex polymerase chain reaction using two primer pairs, namely F1 (5'-GCCAGCCCCCTGATGGGGGCGACACTCCAC-3') and R1 (5'-GCCTATTGGCCTGGAGTGTTTAGCTC-3'), and F2 (5'-GCAGAAAGCGTCTAGCCATGGCGT-3') and R2 (5'-CCAGCGGGGYCGGGCVYGAGACA-3') with the Platinum™ SuperFi DNA polymerase (Thermo Fisher Scientific). Amplified HCV genome cDNA was measured using a Qubit 3.0 fluorometer, to check the quality of the amplified fragments.

### Next-generation sequencing (NGS)

Amplified cDNAs were subject to full-length HCV genome sequencing by H+ proton captured direct-sequencing using an Ion Proton instrument (Thermo fisher scientific). Full-length cDNA was digested with restriction enzymes for 15 min, and fragments were purified with AMPure XP beads. Purified sequencing libraries were ligated to sequence adaptor oligonucleotides and molecular barcodes using the IonXpress Plus gDNA fragment kit (Thermo Fisher Scientific). After library construction, 100 picomoles of the library reads were subject to amplification by emulsion PCR with Hi-Q chemistry using the Ion One Touch 2 instrument and NGS by Ion Torrent semiconductor sequencing with the Ion PI™ chip.

### Identification of genome variants

Mapping the sequence reads to the HCV genome and identification of variants were performed using an in-house computational pipeline based on the Nextflow scripting, which included nucleotide base correction by the k-mer method using the Pollux program, HCV-1b mapping (Con1 [7], HCV-J [8], HCV-BK [9]) by the Burrows-Wheeler Transform algorithm using the BWA and Bowtie2 programs, genome variant detection by the haplotype-based method using FreeBayes program, variant normalization using BCFtools, and variant filtering using VCFtools. FreeBayes detection parameters were set as follows: “-F 0.02 -q 15 -m 20 -U 10 -z 1.0–4 -C 5—min-coverage 6—report-genotype-likelihood-max—dont-left-align-indels,” and VCFtools filtering parameters were set as follows: “QUAL > 0 & DP > 50 & SAF > 50 & SAR > 50.” Moreover, strand bias exclusion parameters were as follows: “SAR/SAF > 0.025 & SAR/SAF < 44.” After variant calling, all the variants detected were incorporated into one VCF file, and functional variants were annotated using the ANNOVAR program. For the machine-learning approach, functional variants with amino acid substitutions and frameshift insertions/deletions were used in basic statistic and machine-learning analysis.

### Machine-learning analysis and statistics

To evaluate the machine-learning approach to finding the optimized combination of functional variants in relation to SVR status following DAA therapy, nine machine-learning methods, including support vector machine (SVM), Random forest (RF), Gradient boost machine (GBM), naive Bayesian (NB), k-nearest neighbor (KNN), Logistic regression (LR), Multi-layer perceptron (Neural network, NN), Flexible discriminant analysis (FDA), and Decision tree (DT) were selected using caret and caret Ensemble packages and R Statistics. All learning algorithms used the binary classification of SVR *vs*. non-SVR, and genotype calling of HCV variants identified 0, 1, and 2 as a no-variant calls, while heterozygous variant calls were based on a >5% fraction of mutant alleles, and homozygous variant calls were based on a >90% fraction of mutant alleles. The Student’s T-test, implemented in R, was applied to analyze differences in the number of variants in the SVR and non-SVR groups.

Machine-learning was performed based on a training data set (reflecting 57 SVR and 27 non-SVR HCV genomes) and a validation-data set (52 SVR and 35 non-SVR genomes) using a random sampling technique. Machine-learning was analyzed by inputting only sequence data. The generalization performance of the machine-learning algorithms was evaluated using accuracy, kappa, positive predictive value (PPV), and negative predictive value (NPV) measures. Additionally, the similarity of selected important variables was evaluated by correlation matrix using the corrplot package of R.

## Results

### Whole HCV genome sequencing and basic statistics

**Fig 2:**
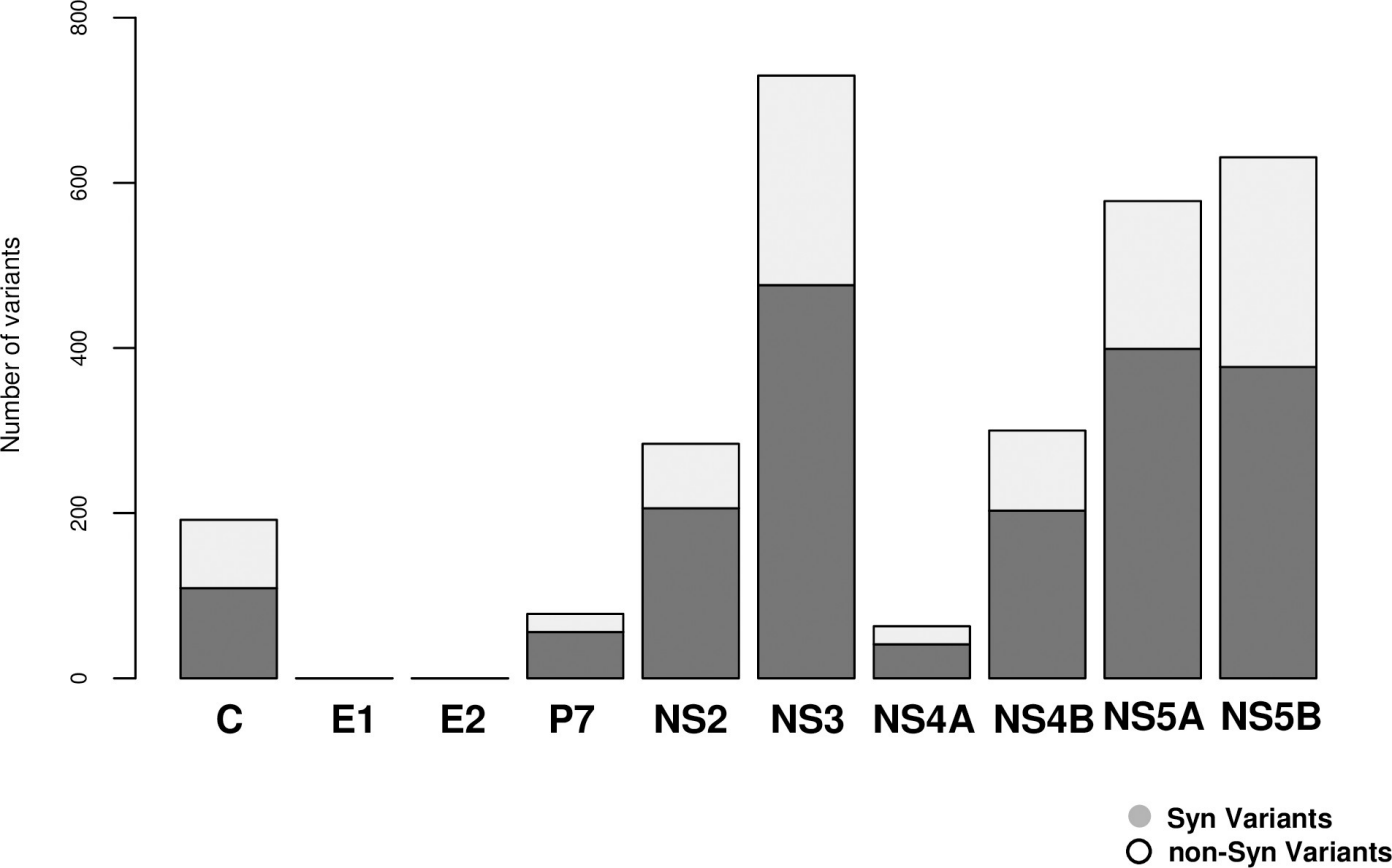
HCV genomic variants (Gene region).

**Fig 3:**
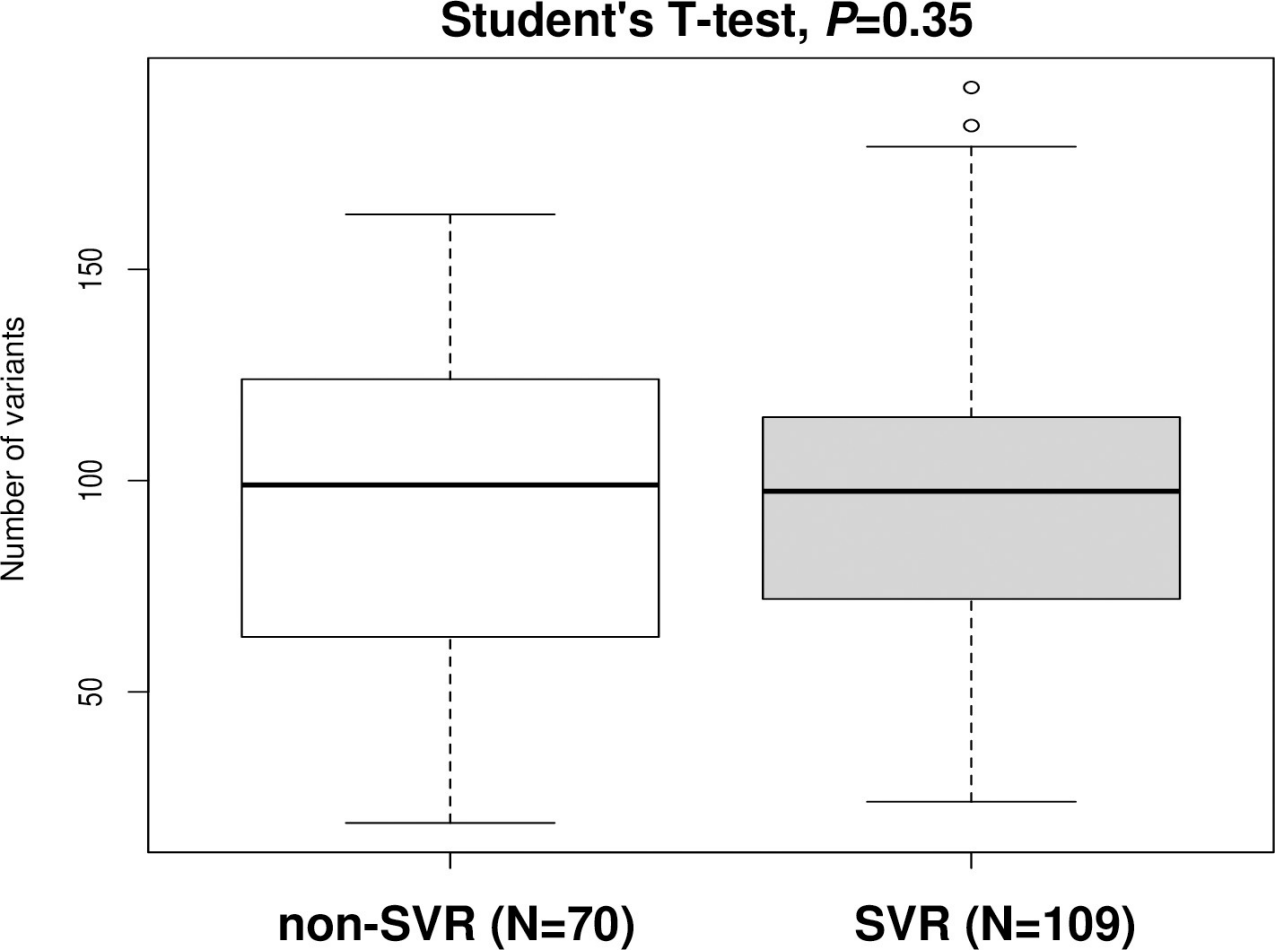
HCV genomic variants (SVR vs. non-SVR).

Sequencing read depth was > 10,000 for all specimens, and 3,461 variants (mean, 188.2 variants/subject; range, 50–341) were detected by our in-house pipeline. Finally, 1,867 variants (mean, 96.15 variants/subject; range, 19–193) that resulted in amino acid changes, and/or had frameshift, stop-gained, or loss-of-function mutations were subject to further analysis. Several mutations were found in NS3, NS5A, and NS5B (Fig 2). Functional variants were equally distributed across each of the HCV genes with an approximately 0.6 times decrement compared with non-functional variants (Fig 2). The distribution ratio of variants for each HCV gene was 0.65% (range, 0.57%–0.73%). No significant difference was observed in the number of functional variants between specimens from SVR patients (mean 94.06; range, 19–163) and non-SVR patients (mean 99.7; range, 24–193) (Student’s T-test, p = 0.35, Fig 3). In the association analysis, 21 variants came out as significant in the LR analysis (FDR < 0.05), including amino acid-changing functional variants of L28 located in NS5A gene.

### Machine learning-based approaches to finding an optimized combination of variants

**Fig 4:**
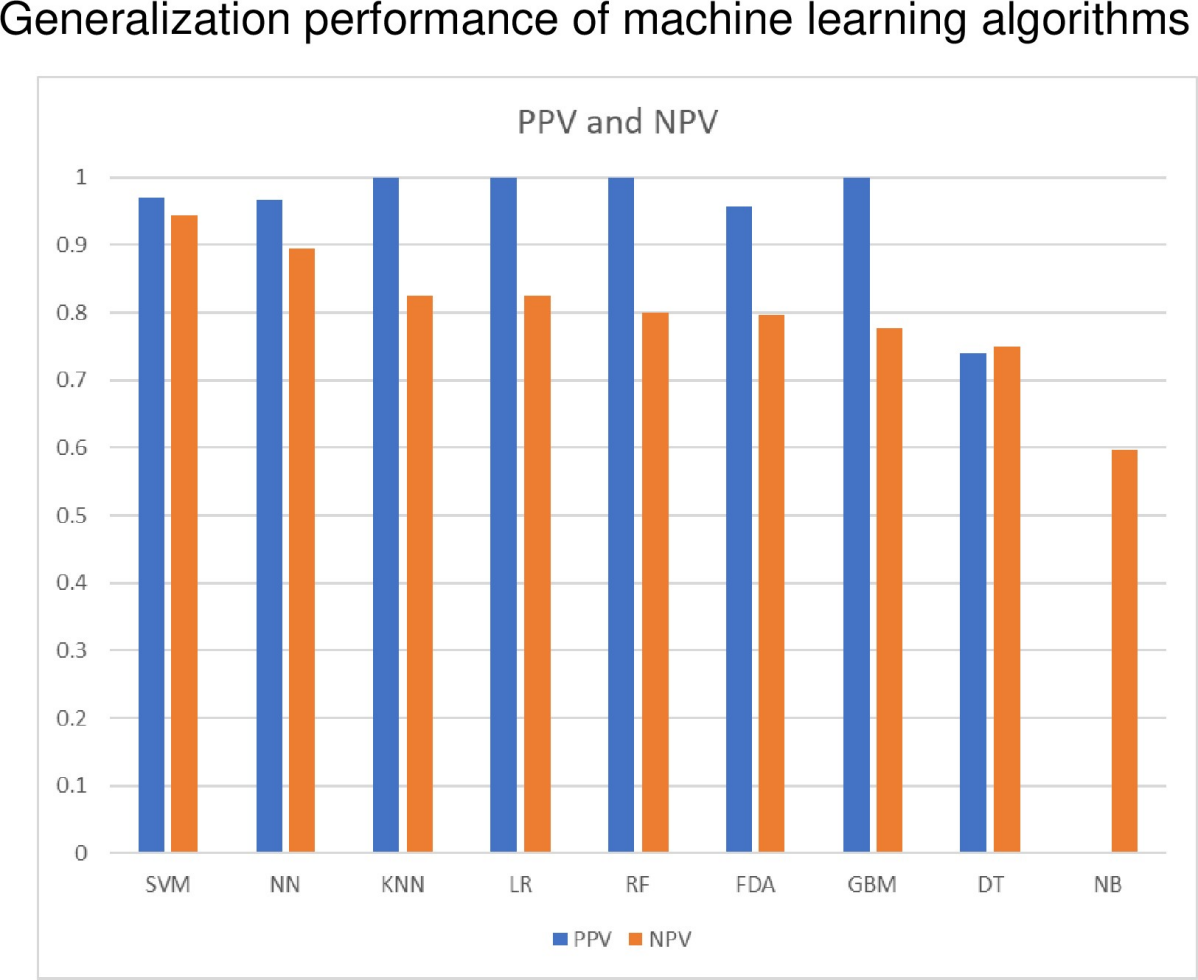
Generalization performance of machine-learning algorithms. SVM: Support vector machine, NN: Neural network, RF: Random forest, LR: Logistic regression, GBM: Gradient boost machine, KNN: K-nearest neighbor, FDA: Flexible discriminant analysis, DT: Decision tree, NB: naive Bayesian.

**Fig 5:**
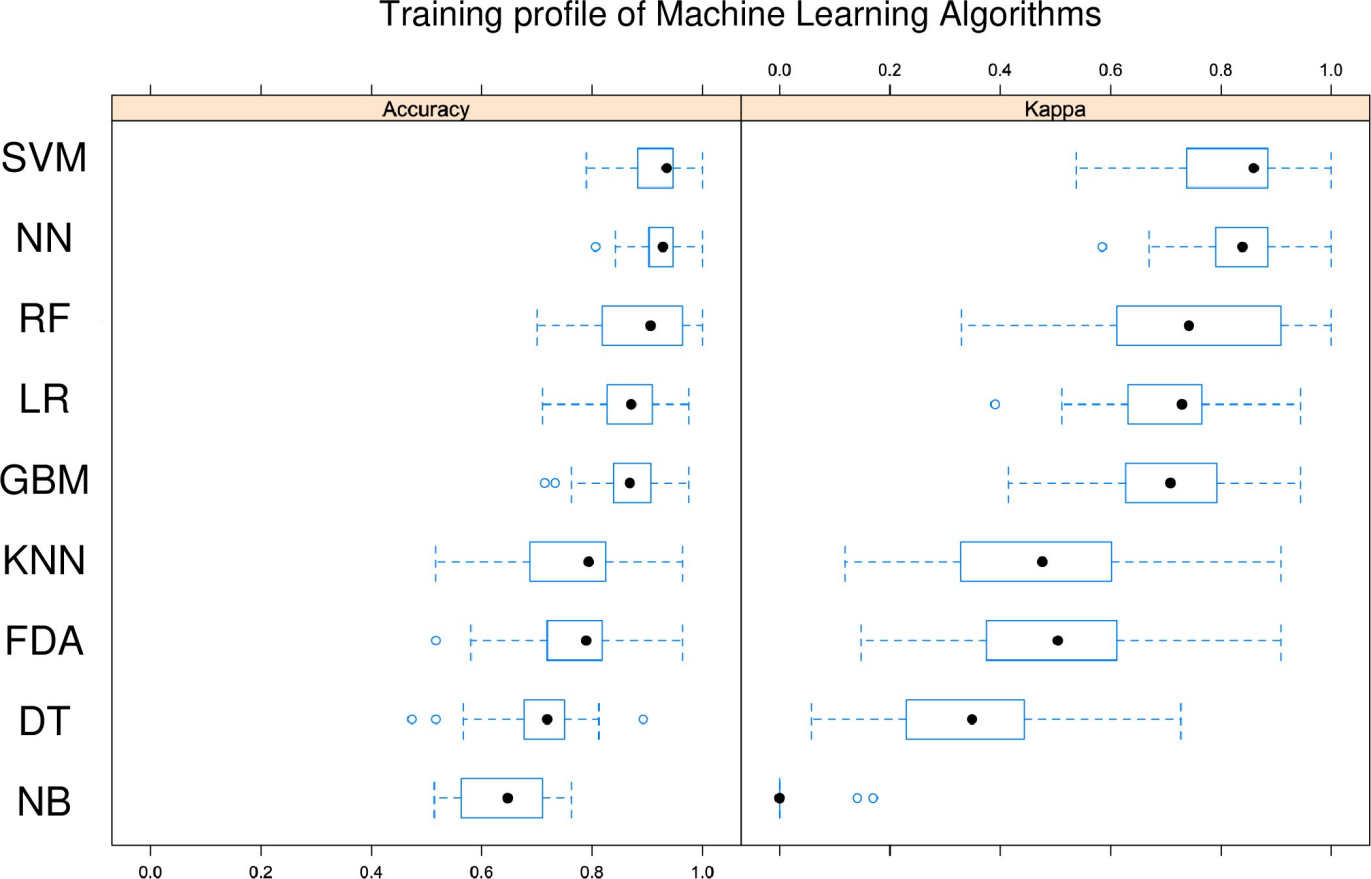
Training profile of machine-learning algorithms. PPV: Positive predictive value, NPV: Negative predictive value, SVM: Support vector machine, NN: Neural network, KNN: K-nearest neighbor, LR: Logistic regression, RF: Random forest, FDA: Flexible discriminant analysis, GBM: Gradient boost machine, DT: Decision tree, NB: Naive Bayesian.

**Fig 6:**
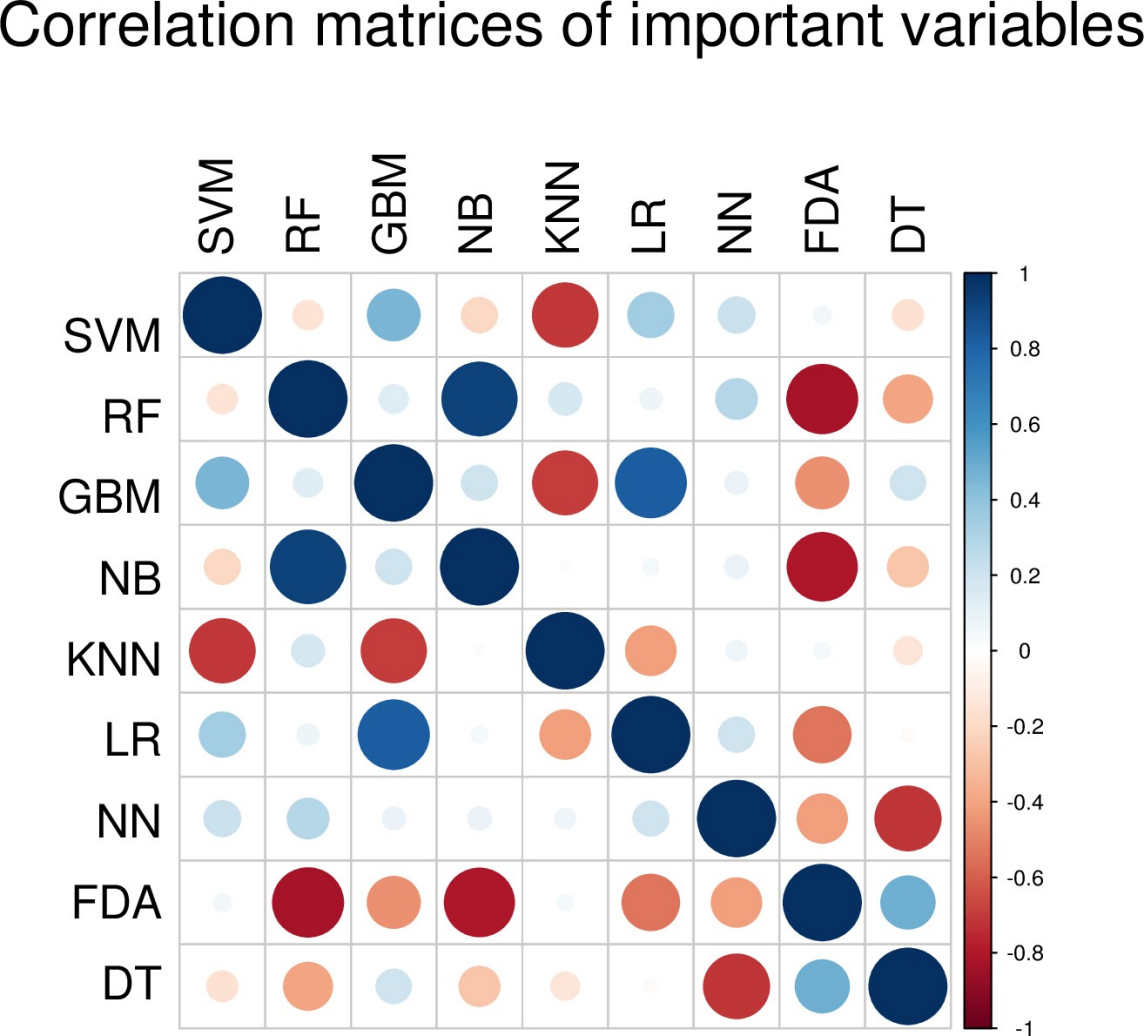
Correlation matrices of important variables. SVM: Support vector machine, RF: Random forest, GBM: Gradient boost machine, NB: Naive Bayesian, KNN: K-nearest neighbor, LR: Logistic regression, NN: Neural network, FDA: Flexible discriminant analysis, DT: Decision tree.

In this study, the most accurate learning method was the SVM algorithm (Table 2 and Fig 4). The second-most accurate learning algorithm was NN. Unfortunately, the DT and NB algorithms could not be fitted with our data because of low accuracy (< 0.8). Notably, only SVM had a generalization performance > 0.9 as measured by PPV and NPV. Some learning algorithms scored > 0.9 in PPV but < 0.8 in NPV (Table 2 and Fig 4). The SVM algorithm also had a kappa score > 0.9, indicating the optimum reproducibility of generalized discriminant performance, while the kappa score for NN was 0.82 (Fig 5). The balanced accuracy scores for the SVR and non-SVR groups were also statistically higher for SVM and NN compared with the other algorithms (Fig 4). Importantly, the variables for the SVM were weakly similar to those of the GBM, LR and NN algorithms, in contrast to the RF, KNN and DT algorithms (Fig 6).

**Table 2:** Performance evaluation of machine learning algorithms.

Machine learning algorithms		Number of Features	Training accuracy	Validation accuracy	Kappa	F Score	Sensitivity	Specificity	Significance
Support Vector Machine	SVM	81	0.937	0.954	0.903	0.941	0.914	0.980	**
Multi-layer Perceptron	NN	3 layers	0.932	0.919	0.828	0.892	0.828	0.980	*
K-Nearest Neighbor	KNN	7	0.797	0.873	0.722	0.813	0.685	1	
Logistic Regression	LR	25	0.848	0.873	0.722	0.813	0.685	1	
Random Forest	RF	1107	0.847	0.85	0.669	0.771	0.628	1	
Flexible Discriminant Analysis	FDA	8	0.744	0.839	0.645	0.758	0.628	0.980	
Gradient Boosting Machine	GBM	47	0.853	0.827	0.614	0.727	0.571	1	
Decision Tree	DT	4	0.74	0.747	0.453	0.645	0.571	0.865	
Naive Bayesian	NB	2161	0.5	0.597	0	-	0	1	

** Sensitivity > 0.90 and specificity > 0.90.

* Sensitivity > 0.80 and specificity > 0.80.

With regard to identifying important predictor variables of functional variants with amino acid changes, L28, Q80, and D168, which are known as resistance-associated substitutions (RAS) variants and located in the NS5A and NS3 genes were identified as components of important predictor variables in SVM. The L28 amino acid-changing variant was also identified as significant in the LR single-point variant analysis (S1 Table).

## Discussion

In this study, it was possible to evaluate all variants by whole genome sequencing of HCV using NGS technology. In the prediction model using the training data set, the correct answer rate was 90% or higher by both SVM and NN. Also in the prediction model with the validation-data set, the correct answer rate was 90% or higher by both SVM and NN; however, the accuracy rate of SVM was the best at 95.4% (kappa statistic 0.90, F-Values 0.94). This is the first report to construct a machine learning-based prediction model of DAA therapeutic effect using whole genome HCV variant data.

We were able to sequence the entire HCV genome using two primer pairs [10,11] when synthesizing cDNA by RT-PCR. Furthermore, in order not to introduce bias into the HCV 1b reference, the original HCV 1b reference was constructed from the common genomic nucleotide sequences of Con1; AJ238799, HCV-J; D90208, HCV-BK; and M95335 [12,13]. The number of HCV genomic variants did not differ significantly between the SVR and non-SVR groups (Fig 3). Several mutations were found in NS3, NS5A, and NS5B (Fig 2).

In this study, nine machine-learning algorithms (SVM, RF, GBM, NB, KNN, LR, NN, FDA, and DT) were applied to the training data set (SVR, n = 57; non-SVR, n = 29) and validation-data set (SVR, n = 52; non-SVR, n = 35) by random sampling. In the prediction model using the training data set, a good prediction model with a accuracy rate of 90% or more was obtained by both SVM and NN (Table 2). The HCV genomic variants identified by the prediction model tended to be those that have been previously reported to be associated with successful DAA, such as the NS5A p.L28M mutation [14] and the p.R566H mutation [15]. The number of predictors provided by SVM, NN, KNN, LR, RF, FDA, GBM, DT, and NB were 81, 3, 7, 25, 1107, 8, 47, 4, and 2161, respectively. NB, KNN, and NN were prediction models using all variants as predictors. All of the learning algorithms provided prediction models that identified known variants typically associated with successful DAA treatment [16]. In the generalization performance evaluation of the prediction model using the validation-data set, the accuracy rate of SVM was 95.4% (kappa coefficient 0.90, F-value 0.94), indicating a very high discriminative ability and high reproducibility. Also for NN, KNN, LR, and RF the accuracy rate was 85% or more, indicating a high generalization performance. SVMs with particularly high generalization ability have been developed as learning algorithms that combine kernel methods with neural network algorithms. Furthermore, it has the advantage that it can be used regardless of (non)linear regression model, and there is little influence of over-learning. Currently, NN is often used instead of SVM, but our study showed that SVM had a higher accuracy rate than NN. RF and GBM, which are examples of ensemble learning, generally also have a high accuracy rate and a high degree of coincidence and are suitable for machine-learning algorithms combining genome analysis and clinical data. However, it is necessary to pay attention to over-training in ensemble learning, and it is necessary to carefully evaluate the parameter values individually for each learning algorithm when making prediction model determinations. It has been reported that the difficulty of achieving successful DAA treatment is proportionate to the amount of RAS mutations in NS5A genes (p.L31M and p.Y93H) [17]. In the present study, machine learning-based algorithms were trained on a combination of known variants of RAS and other variants.

DAA treatment has improved dramatically, and, currently, Ledipasvir/Sofosbuvir [18], Elbasvir/Grazoprevir [19], or Glecaprevir/Pibrentasvir [20] can be used in Japan to enable most patients to eliminate the virus. In addition, Sofosbuvir/Velpatasvir can be used for patients with decompensated cirrhosis [21]. The DAAs used in this study were Daclatasvir/Asunaprevir, Ledipasvir/Sofosbuvir, and Ledipasvir/Sofosbuvir + Ribavirin. The initial DAA treatment with Daclatasvir/Asunaprevir produced lower SVR rates in this study than those found in the most recent reports [22]. This pre-prediction model seems to depend on the DAA used and should be considered for each new DAA going forward. Currently, Daclatasvir/Asunaprevir is excluded from Japanese treatment guidelines, which may reduce the need for this predictive model. However, this preliminary prediction model can inform on similar applications within the medical field, and we believe that the method of identifying variants of a virus based on whole genome data, as well as using machine-learning to create a predictive system for therapeutic effects, can be applied to identify optimal treatments for other viral infections and cancer.

Conclusively, all variants of HCV whole-genome sequences could be evaluated, and the accuracy rate of SVM was 95.4% (kappa statistic, 0.90; F-Values, 0.94), which was the best model among the nine models tested in this study. Prior to treatment, predicting the SVR to identify difficult-to-treat patients, who cannot eliminate the HCV using DAAs alone, may be effective in avoiding the emergence of a resistant virus and reducing medical costs. Our approach to genomic analysis and the construction of a predictive model by machine-learning may inform similar approaches aiming to identify the optimal treatment for other viral infections and cancers as well.

## Supporting information

S1 Table Important predictor variables. (DOCX)
