# Supplementary material for: A machine learning-based treatment prediction model using whole genome variants of hepatitis C virus
Source: PLoS One. 2020 Nov 5;15(11):e0242028. doi: 10.1371/journal.pone.0242028 (PMC7644079; doi:10.1371/journal.pone.0242028)
Supplement: S1 Table — (DOCX) [file pone.0242028.s001.docx]

**Supplementary Table 1**

**Important predictor variables**
